# Assessment of N95 respirator decontamination and re-use for SARS-CoV-2

**DOI:** 10.1101/2020.04.11.20062018

**Published:** 2020-04-24

**Authors:** Robert J. Fischer, Dylan H. Morris, Neeltje van Doremalen, Shanda Sarchette, M. Jeremiah Matson, Trenton Bushmaker, Claude Kwe Yinda, Stephanie N. Seifert, Amandine Gamble, Brandi N. Williamson, Seth D. Judson, Emmie de Wit, James O. Lloyd-Smith, Vincent J. Munster

**Affiliations:** 1 National Institute of Allergy and Infectious Diseases, Hamilton, MT; 2 Princeton University, Princeton, NJ; 3 University of California, Los Angeles, Los Angeles, CA; 4 University of Washington, Seattle, WA

Dear editor,

The unprecedented pandemic of COVID-19 has created worldwide shortages of personal protective equipment, in particular respiratory protection such as N95 respirators(1). SARS-CoV-2 transmission is frequently occurring in hospital settings, with numerous reported cases of nosocomial transmission highlighting the vulnerability of healthcare workers(2). The environmental stability of SARS-CoV-2 underscores the need for rapid and effective decontamination methods. In general, N95 respirators are designed for single use prior to disposal. Extensive literature is available for decontamination procedures for N95 respirators, using either bacterial spore inactivation tests, bacteria or respiratory viruses (e.g. influenza A virus)(3–6). Effective inactivation methods for these pathogens and surrogates include UV, ethylene oxide, vaporized hydrogen peroxide (VHP), gamma irradiation, ozone and dry heat(3–7). The filtration efficiency and N95 respirator fit has typically been less well explored, but suggest that both filtration efficiency and N95 respirator fit can be affected by the decontamination method used(7, 8). For a complete list of references see supplemental information.

Here, we analyzed four different decontamination methods – UV radiation (260 – 285 nm), 70°C dry heat, 70% ethanol and vaporized hydrogen peroxide (VHP) – for their ability to reduce contamination with infectious SARS-CoV-2 and their effect on N95 respirator function. For each of the decontamination methods, we compared the normal inactivation rate of SARS-CoV-2 on N95 filter fabric to that on stainless steel, and we used quantitative fit testing to measure the filtration performance of the N95 respirators after each decontamination run and 2 hours of wear, for three consecutive decontamination and wear sessions (see supplemental information). VHP and ethanol yielded extremely rapid inactivation both on N95 and on stainless steel (Figure 1A). UV inactivated SARS-CoV-2 rapidly from steel but more slowly on N95 fabric, likely due its porous nature. Heat caused more rapid inactivation on N95 than on steel; inactivation rates on N95 were comparable to UV.

Quantitative fit tests showed that the filtration performance of the N95 respirator was not markedly reduced after a single decontamination for any of the four decontamination methods (Figure 1B). Subsequent rounds of decontamination caused sharp drops in filtration performance of the ethanol-treated masks, and to a slightly lesser degree, the heat-treated masks. The VHP and UV treated masks retained comparable filtration performance to the control group after two rounds of decontamination, and maintained acceptable performance after three rounds.

Taken together, our findings show that VHP treatment exhibits the best combination of rapid inactivation of SARS-CoV-2 and preservation of N95 respirator integrity, under the experimental conditions used here (Figure 1C). UV radiation kills the virus more slowly and preserves comparable respirator function. 70°C dry heat kills with similar speed to UV and is likely to maintain acceptable fit scores for two rounds of decontamination. Ethanol decontamination is not recommended due to loss of N95 integrity, echoing earlier findings^5^.

All treatments, particularly UV and dry heat, should be conducted for long enough to ensure that a sufficient reduction in virus concentration has been achieved. The degree of required reduction will depend upon the degree of initial virus contamination. Policymakers can use our estimated decay rates together with estimates of real-world contamination to choose appropriate treatment durations (see supplemental information).

Our results indicate that N95 respirators can be decontaminated and re-used in times of shortage for up to three times for UV and HPV, and up to two times for dry heat. However, utmost care should be given to ensure the proper functioning of the N95 respirator after each decontamination using readily available qualitative fit testing tools and to ensure that treatments are carried out for sufficient time to achieve desired risk-reduction. It will therefore be critical that FDA, CDC and OSHA guidelines with regards to fit testing, seal check and respirator re-use are followed(9, 10).

## Supplementary Material

1

## Figures and Tables

**Figure 1.**
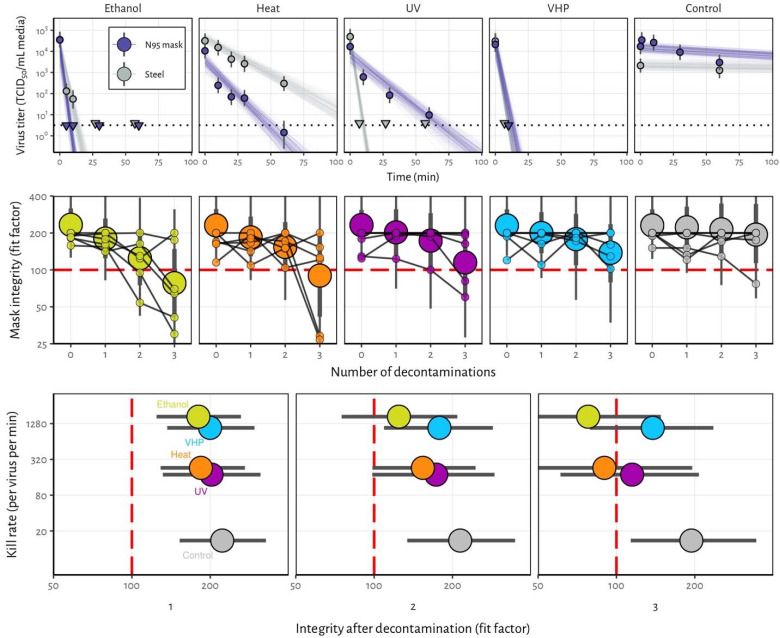
Decontamination of SARS-CoV-2 by four different methods. **A)** SARS-CoV-2 on N95 fabric and stainless steel surface was exposed to UV, 70 °C dry heat, 70% ethanol and vaporized hydrogen peroxide (VHP). 50 μl of 10^5^ TCID_50_/mL of SARS-CoV was applied on N95 and stainless steel (SS). Samples were collected at indicted time-points post exposure to the decontamination method for UV, heat and ethanol and after 10 minutes for VHP. Viable virus titer is shown in TCID_50_/mL media on a logarithmic scale. All samples were quantified by end-point titration on Vero E6 cells. Plots show estimated mean titer across three replicates (circles and bars show the posterior median estimate of this mean and a 95% credible interval). Time-points with no positive wells for any replicate are plotted as triangles at the approximate single-replicate detection limit of the assay (LOD, see Appendix for discussion) to indicate that a range of sub-LOD values are plausible. Steel points at the LOD are offset slightly up and to the left to avoid overplotting. Lines show predicted decay of virus titer over time (lines; 50 random draws per replicate from the joint posterior distribution of the exponential decay rate, i.e. negative of the slope, and intercept, i.e. initial virus titer). Black dotted line shows approximate LOD: 10^0.5^ TCID_50_/mL media. **B)** Mask integrity. Quantitative fit testing results for all the decontamination methods after decontamination and 2 hours of wear, for three consecutive runs. Data from six individual replicates (small dots) for each treatment are shown in addition to an estimated median fit factor (large dots), an estimated 68% range of fit factors (thick bars) and an estimated 95% range (thin bars). Fit factors are a measure of filtration performance: the ratio of the concentration of particles outside the mask to the concentration inside. The measurement machine reports value up to 200. A minimal fit factor of 100 (red dashed line) is required for a mask to pass a fit test. **C)** SARS-CoV-2 decontamination performance. Kill rate (y-axis), versus mask integrity after decontamination (x-axis; point represents estimated median, bar length represents estimated 68% range). The three panels report mask integrity after one, two or three decontamination cycles.
