# Supplementary material for: Assessment of N95 respirator decontamination and re-use for SARS-CoV-2
Source: medRxiv. 2020 Apr 24:2020.04.11.20062018. Preprint. [Version 2] doi: 10.1101/2020.04.11.20062018 (PMC7217083; doi:10.1101/2020.04.11.20062018)
Supplement: 1 [file NIHPP2020.04.11.20062018-supplement-1.pdf]

medRxiv preprint doi: https://doi.org/10.1101/2020.04.11.20062018.this version posted April 24, 2020. The copyright holder for this preprint (which was not certified by peer review) is the author/funder, who has granted medRxiv a license to display the preprint in perpetuity. This article is a US Government work. It is not subject to copyright under 17 USC 105 and is also made available for use under a CC0 license.

Table of contents: page 1

Supplemental methods page 2
Supplemental table page 10
Code and data availability page 10
Acknowledgements page 11
Supplemental references page 11

medRxiv preprint doi: https://doi.org/10.1101/2020.04.11.20062018.this version posted April 24, 2020. The copyright holder for this preprint (which was not certified by peer review) is the author/funder, who has granted medRxiv a license to display the preprint in perpetuity. This article is a US Government work. It is not subject to copyright under 17 USC 105 and is also made available for use under a CC0 license.

Supplemental methods

Short literature review on mask decontamination

The COVID-19 pandemic has highlighted the necessity for large-scale decontamination procedures for personal protective equipment (PPE), in particular N95 respirator masks(1). SARS-CoV-2 has frequently been detected on PPE of healthcare workers(11). The environmental stability of SARS-CoV-2 underscores the need for rapid and effective decontamination methods(12). Extensive literature is available for decontamination procedures for N95 respirators, using either bacterial spore inactivation tests, bacteria or respiratory viruses (e.g. influenza A virus)(3-6, 9, 13-15). Effective inactivation methods for these pathogens and surrogates include UV, ethylene oxide, vaporized hydrogen peroxide (VHP), gamma irradiation, ozone and dry heat(4, 5, 7, 9, 14-16). The filtration efficiency and N95 respirator fit has typically been less well explored, but suggest that both filtration efficiency and N95 respirator fit can be affected by the decontamination method used(7, 8). It will therefore be critical that FDA, CDC and OSHA guidelines with regards to fit testing, seal check and respirator re-use are followed(9, 17-20).

Laboratory experiments

Viruses and titration

HCoV-19 nCoV-WA1-2020 (MN985325.1) was the SARS-CoV-2 strain used in our comparison(21). Virus was quantified by end-point titration on Vero E6 cells as described previously(22). Virus titrations were performed by end-point titration in Vero E6 cells. Cells were inoculated with 10-fold serial dilutions in four-fold of samples taken from N95 mask and stainless steel surfaces (see below). One hour after inoculation of cells, the inoculum was removed and replaced with 100 µl (virus titration) DMEM (Sigma-Aldrich) supplemented with 2% fetal bovine serum, 1 mM L-glutamine, 50 U/ml penicillin and 50 µg/ml streptomycin. Six days after inoculation, cytopathogenic effect was scored and the $TCID_{50}$ was calculated (see below). Wells presenting cytopathogenic effects due to media toxicity

medRxiv preprint doi: https://doi.org/10.1101/2020.04.11.20062018.this version posted April 24, 2020. The copyright holder for this preprint (which was not certified by peer review) is the author/funder, who has granted medRxiv a license to display the preprint in perpetuity. This article is a US Government work. It is not subject to copyright under 17 USC 105 and is also made available for use under a CC0 license.

(e.g., due to the presence of ethanol or hydrogen peroxide) rather than viral infection were removed from the titer inference procedure.

N95 and stainless steel surface

N95 material discs were made by punching 9/16” (15 mm) fabric discs from N95 respirators, AOSafety N9504C respirators (Aearo Company Southbridge, MA). The stainless steel 304 alloy discs were purchased from Metal Remnants (https://metalremnants.com/) as described previously. 50 µL of SARS-CoV-2 was spotted onto each disc. A 0 time-point measurement was taken prior to exposing the discs to the disinfection treatment. At each sampling time-point, discs were rinsed 5 times by passing the medium over the stainless steel or through the N95 disc. The medium was transferred to a vial and frozen at -80°C until titration. All experimental conditions were performed in triplicate.

Decontamination methods

Ultraviolet light. Plates with fabric and steel discs were placed under an LED high power UV germicidal lamp (effective UV wavelength 260-285nm) without the titanium mesh plate (LEDi2, Houston, Tx) 50 cm from the UV source. At 50 cm the UVC power was measured by the manufacturer at 550 µW/cm^2 . Plates were removed at 10, 30 and 60 minutes and 1 mL of cell culture medium added. The energy the discs were exposed to at 10, 30 and 60 min is 0.33 J/cm^2, 0.99 J/cm^2, and 1.98 J/cm^2 respectively. While the CDC has no specific recommendations on the minimum dose, they do report that a 1 J/cm2 dose can reduce tested viable viral loads by 99.9%[4].

Heat treatment. Plates with fabric and steel discs were placed in a 70°C oven. Plates were removed at 10, 20, 30 and 60 minutes and 1 mL of cell culture medium added.

70% ethanol. Fabric and steel discs were placed into the wells of one 24 well plate per time-point and sprayed with 70% ethanol to saturation. The plate was tipped to near vertical and 5 passes of ethanol were

medRxiv preprint doi: https://doi.org/10.1101/2020.04.11.20062018.this version posted April 24, 2020. The copyright holder for this preprint (which was not certified by peer review) is the author/funder, who has granted medRxiv a license to display the preprint in perpetuity. This article is a US Government work. It is not subject to copyright under 17 USC 105 and is also made available for use under a CC0 license.

sprayed onto the discs from approximately 10 cm. After 10 minutes, 1 mL of cell culture medium was added.

VHP. Plates with fabric and steel discs were placed into a Panasonic MCO-19AIC-PT (PHC Corp. of North America Wood Dale, IL) incubator with VHP generation capabilities and exposed to hydrogen peroxide (approximately 1000 ppm). The exposure to VHP was 7 minutes, after the inactivation of the hydrogen peroxide, the plate was removed and 1 mL of cell culture medium was added.

Control. Plates with fabric and steel discs and steel plates were maintained at 21-23°C and 40% relative humidity for up to four days. After the designated time-points, 1 mL of cell culture medium was added.

N95 mask integrity testing

N95 Mask (3M™ Aura™ Particulate Respirator 9211+/37193) integrity testing after 2 hours of wear and decontamination, for three consecutive rounds, was performed for a total of 6 times for each decontamination condition and control condition. Masks were worn by subjects and integrity was quantitatively determined using the Portacount Respirator fit tester (TSI, 8038) with the N95 companion component, following the modified ambient aerosol condensation nuclei counter quantitative fit test protocol approved by the OSHA[18]. Subjects were asked to bend over for 40 seconds, talk for 50 seconds, move head from side-to-side for 50 seconds, and move head up-and-down for 50 seconds whilst aerosols on inside and outside of mask were measured. By convention, this fit test is passed when the final score is ≥100. For the N95 integrity testing, a Honeywell Mistmate humidifier (cat#HUL520B) was used for particle generation.

Statistical analyses

In the model notation that follows, the symbol ~ denotes that a random variable is distributed according to the given distribution. Normal distributions are parametrized as Normal(mean, standard deviation). Positive-constrained normal distributions (“Half-Normal”) are parametrized as Half-

medRxiv preprint doi: https://doi.org/10.1101/2020.04.11.20062018.this version posted April 24, 2020. The copyright holder for this preprint (which was not certified by peer review) is the author/funder, who has granted medRxiv a license to display the preprint in perpetuity. This article is a US Government work. It is not subject to copyright under 17 USC 105 and is also made available for use under a CC0 license.

Normal(mode, standard deviation). Normal distributions truncated to the interval [0, 1] are parameterized as TruncNormal(mode, standard deviation).

We use <Distribution Name>CDF(x | parameters) and <Distribution Name>CCDF to denote the cumulative distribution function and complementary cumulative distribution functions of a probability distribution, respectively. So for example NormalCDF(5 | 0, 1) is the value of the Normal(0, 1) cumulative distribution function at 5.

We use logit(x) and invlogit(x) to denote the logit and inverse logit functions, respectively:

$$\text{logit}(x) = \ln \frac{x}{1 - x} \qquad (1)$$

$$\text{invlogit}(x) = \frac{e^x}{1 + e^x} \qquad (2)$$

Mean titer inference

We inferred mean titers across sets of replicates using a Bayesian model. The $\log_{10}$ titers v_{ijk} (the titer for the sample from replicate k of time-point j of experiment i) were assumed to be normally distributed about a mean μ_{ij} with a standard deviation σ. We placed a very weakly informative normal prior on the $\log_{10}$ mean titers μ_{ij}:

$$\mu_{ij} \sim \text{Normal}(3, 3) \qquad (3)$$

We placed a weakly informative normal prior on the standard deviation:

$$\sigma \sim \text{Normal}(0, 0.5) \qquad (4)$$

We then modeled individual positive and negative wells for sample ijk according to a Poisson single-hit model(23). That is, the number of virions that successfully infect cells in a given well is Poisson distributed with mean:

medRxiv preprint doi: https://doi.org/10.1101/2020.04.11.20062018.this version posted April 24, 2020. The copyright holder for this preprint (which was not certified by peer review) is the author/funder, who has granted medRxiv a license to display the preprint in perpetuity. This article is a US Government work. It is not subject to copyright under 17 USC 105 and is also made available for use under a CC0 license.

$$V = \ln(2)\, 10^{v} \qquad (5)$$

where v is the $\log_{10}$ virus titer in $TCID_{50}$, where v is the $\log_{10}$ virus titer in $TCID_{50}$, and the well is infected if at least one virion successfully infects a cell. The value of the mean derives from the fact that our units are $TCID_{50}$; the probability of infection at $v = 0$, i.e. 1 $TCID_{50}$, is equal to $1 – e^{- \ln(2) \times 1} = 0.5$.

Let Y_{ijkdl} be a binary variable indicating whether the lth well of dilution factor d (expressed as $\log_{10}$ dilution factor) of sample ijk was positive (so $Y_{ijkdl} = 1$ if the well was positive and 0 otherwise), which will occur as long as at least one virion successfully infects a cell.

It follows from (5) that the conditional probability of observing $Y_{ijkdl} = 1$ given a true underlying titer $\log_{10}$ titer v_{ijk} is given by:

$$L(Y_{ijkdl} = 1 \mid v_{ijk}) = 1 - e^{-\ln(2) \times 10^{x}} \qquad (6)$$

Where

$$x = v_{ijk} - d \qquad (7)$$

is the expected concentration, measured in $\log_{10}$ $TCID_{50}$, in the dilute sample. This is simply the probability that a Poisson random variable with mean $(– \ln(2) \times 10^{x})$ is greater than 0. Similarly, the conditional probability of observing $Y_{ijkdl} = 0$ given a true underlying titer $\log_{10}$ titer v_{ijk} is given by:

$$L(Y_{ijkdl} = 0 \mid v_{ijk}) = e^{-\ln(2) \times 10^{x}} \qquad (8)$$

which is the probability that the Poisson random variable is 0.

This gives us our likelihood function, assuming independence of outcomes across wells.

Virus inactivation regression

medRxiv preprint doi: https://doi.org/10.1101/2020.04.11.20062018.this version posted April 24, 2020. The copyright holder for this preprint (which was not certified by peer review) is the author/funder, who has granted medRxiv a license to display the preprint in perpetuity. This article is a US Government work. It is not subject to copyright under 17 USC 105 and is also made available for use under a CC0 license.

The durations of detectability depend on the decontamination treatment but also initial inoculum and sampling method, as expected. We therefore estimated the decay rates of viable virus titers using a Bayesian regression analogous to that used in van Doremalen et al., 2020(12). This modeling approach allowed us to account for differences in initial inoculum levels across replicates as well as other sources of experimental noise. The model yields estimates of posterior distributions of viral decay rates and half-lives in the various experimental conditions – that is, estimates of the range of plausible values for these parameters given our data, with an estimate of the overall uncertainty(24).

Our data consist of 10 experimental conditions: 2 materials (N95 masks and stainless steel) by 5 treatments (no treatment, ethanol, heat, UV and VHP). Each has three replicates, and multiple time-points for each replicate. We analyze the two materials separately. For each, we denote by Y_{ijkdl} the positive or negative status (see above) for well l which has dilution d for the titer v_{ijk} from experimental condition i during replicate j at time-point k.

We model each replicate j for experimental condition i as starting with some true initial $\log_{10}$ titer $v_{ij}(0) = v_{ij0}$. We assume that viruses in experimental condition i decay exponentially at a rate λ_i over time t. It follows that:

$$v_{ij}(t) = v_{ij0} - \lambda_i t \quad (9)$$

We use the direct-from-well data likelihood function described above, except that now instead of estimating titer distribution about a shared mean μ_{ij} we estimate λ_i under the assumptions that our observed well data Y_{ijkdl} reflect the titers $v_{ij}(t)$.

Regression prior distributions

We place a weakly informative Normal prior distribution on the initial $\log_{10}$ titers v_{ij0} to rule out implausibly large or small values (e.g. in this case undetectable $\log_{10}$ titers or $\log_{10}$ titers much higher than the deposited concentration), while allowing the data to determine estimates within plausible ranges:

medRxiv preprint doi: https://doi.org/10.1101/2020.04.11.20062018.this version posted April 24, 2020. The copyright holder for this preprint (which was not certified by peer review) is the author/funder, who has granted medRxiv a license to display the preprint in perpetuity. This article is a US Government work. It is not subject to copyright under 17 USC 105 and is also made available for use under a CC0 license.

$$v_{ij0} \sim \text{Normal}(4.5, 2) \qquad (10)$$

We placed a weakly informative Half-Normal prior on the exponential decay rates λ_i:

$$\lambda_i \sim \text{Half-Normal}(0.5, 4) \qquad (11)$$

Our plated samples were of volume 0.1 mL, so inferred titers were incremented by 1 to convert to units of $\log_{10}$ $TCID_{50}$/mL.

Mask integrity estimation

To quantify the decay of mask integrity after repeated decontamination, we used a logit-linear spline Bayesian regression to estimate the rate of degradation of mask fit factors over time, accounting for the fact that fit factors are interval-censored ratios. Fit factors are defined as the ratio of exterior concentration to interior concentration of a test aerosol. They are reported to the nearest integer, up to a maximum readout of 200, but arbitrarily large true fit factors are possible as the mask performance approaches perfect filtration.

We had 6 replicate masks j for each of 5 treatments i (no decontamination, ethanol, heat, UV and VHP). Each mask j was assessed for fit factor at 4 time-points k: before decontamination, and then after 1, 2, and 3 decontamination cycles. We label the control treatment $i = 0$. So we denote by F_{ijk} the fit factor for the jth mask from the ith treatment after k decontaminations (with $k = 0$ for the initial value).

We first converted fit factors F_{ijk} to the equivalent observed filtration rate Y_{ijk} by:

$$Y = 1 - 1/F \qquad (12)$$

Observation model and likelihood function

medRxiv preprint doi: https://doi.org/10.1101/2020.04.11.20062018.this version posted April 24, 2020. The copyright holder for this preprint
(which was not certified by peer review) is the author/funder, who has granted medRxiv a license to display the preprint in perpetuity.
This article is a US Government work. It is not subject to copyright under 17 USC 105 and is also made available
for use under a CC0 license.

We modeled the censored observation process as follows. logit(Y_{ijk}) values are observed with Gaussian error about the true filtration logit(p_{ijk}), with an unknown standard deviation σ_o, and then converted to fit factors, which are then censored:

$$\text{logit}(Y_{ijk}) \sim \text{Normal}(\text{logit}(p_{ijk}), \sigma_o) \qquad (13)$$

Because our reported fit factors are known to be within integer values and right-censored at 200, for $F_{ijk} \geq 200$ we have a conditional probability of observing the data given the parameters of

$$\text{L}(F_{\text{ijk}} \mid p_{ijk}, \sigma_o) = \text{NormalCCDF}(\text{logit}(1 - 1/200) \mid \text{logit}(p_{ijk})\ \sigma_o) \qquad (14)$$

That is, we calculate the probability of observing a value of F greater than or equal to 200 (equivalent a value of Y greater than or equal to 1 – 1/200), given our parameters.

For $1.5 \leq F_{ijk} < 200$, we first calculate the upper and lower bounds of our observation $Y^{+}_{ijk} = 1 - 1 / (F_{\text{ijk}} - 0.5)$ and $Y^{-}_{ijk} = 1 - 1 / (F_{ijk} - 0.5)$. Then:

$$\text{L}(F_{\text{ijk}} \mid p_{ijk}, \sigma_o) = \text{NormalCDF}(\text{logit}(Y^{+}_{ijk}) \mid \text{logit}(p_{ijk})\ \sigma_o) -$$
$$\text{NormalCDF}(\text{logit}(Y^{-}_{ijk}) \mid \text{logit}(p_{ijk})\ \sigma_o) \qquad (15)$$

That is, we calculate the probability of observing a value between Y^{+}_{ijk} and Y^{-}_{ijk}, given our parameters.

medRxiv preprint doi: https://doi.org/10.1101/2020.04.11.20062018.this version posted April 24, 2020. The copyright holder for this preprint (which was not certified by peer review) is the author/funder, who has granted medRxiv a license to display the preprint in perpetuity. This article is a US Government work. It is not subject to copyright under 17 USC 105 and is also made available for use under a CC0 license.

Decay model

We assumed that each mask had some true initial filtration rate p_{ij0}. We assumed that these were logit-normally distributed about some unknown mean mask initial filtration rate p_{avg} with a standard deviation σ_p, that is:

$$\text{logit}(p_{ij0}) \sim \text{Normal}\left(\text{logit}(p_{avg}), \sigma_p\right) \qquad (16)$$

We then assumed that the logit of the filtration rate, logit(p_{ijk}), decreased after each decontamination by a quantity $d_{0k} + d_{ik}$, where d_{0k} is natural degradation during the kth trial in the absence of decontamination (i.e. the degradation rate in the control treatment, $i = 0$), and d_{ik} is the additional degrading effect of the kth decontamination treatment of type $i > 0$). So for $k = 1, 2, 3$ and $i > 0$:

$$\text{logit}(p_{ijk}) = \text{logit}(p_{ij(k-1)}) - (d_{0k} + d_{ik}) + \varepsilon_{ijk} \qquad (17)$$

where ε_{ijk} is a normally-distributed error term with an inferred standard deviation $\sigma_{\varepsilon ik}$ for each treatment and decontamination level.

$$\varepsilon_{ijk} \sim \text{Normal}(0, \sigma_{\varepsilon ik}) \qquad (18)$$

And for the control $i = 0$:

$$\text{logit}(p_{0jk}) = \text{logit}(p_{0j(k-1)}) - d_{0k} + \varepsilon_{0jk} \qquad (19)$$

Model prior distributions

We placed a weakly informative Half-Normal prior on the control degradation rate d_0:

$$d_0 \sim \text{Half-Normal}(0, 0.5) \qquad (20)$$

We placed a weakly informative Half-Normal prior on the non-control degradation rates d_i, $i > 0$:

medRxiv preprint doi: https://doi.org/10.1101/2020.04.11.20062018.this version posted April 24, 2020. The copyright holder for this preprint (which was not certified by peer review) is the author/funder, who has granted medRxiv a license to display the preprint in perpetuity. This article is a US Government work. It is not subject to copyright under 17 USC 105 and is also made available for use under a CC0 license.

$$d_i \sim \text{Half-Normal}(0.25, 0.5) \qquad (21)$$

reflecting the conservative assumption that decontamination should degrade the mask at least somewhat.

We placed a Truncated Normal prior on the mean initial filtration p_{avg}:

$$p_{avg} \sim \text{TruncNormal}(0.995, 0.02) \qquad (22)$$

The mode of 0.995 corresponds to the maximum measurable fit factor of 200. The standard deviation of 0.02 leaves it plausible that some masks could start near or below the minimum acceptable threshold fit factor of 100, which corresponds to a p of 0.99.

We placed weakly informative Half-Normal priors on the logit-space standard deviations σ_p, $\sigma_{\varepsilon ik}$, and σ_o. σ_p reflects variation in individual masks' initial filtration about p_{avg}. The various $\sigma_{\varepsilon ik}$ reflect variation in masks' true degree of degradation between decontaminations about the expected degree of decay, and σ_o reflects noise in the observation process.

$$\sigma_p, \sigma_o \sim \text{Half-Normal}(0, 0.5)$$

$$(23)$$

$$\sigma_{\varepsilon ik} \sim \text{Half-Normal}(0, 0.33)$$

We chose standard deviations less than or equal to 0.5 for these normal hyperpriors because a standard deviation of 1.5 (i.e. 3 σ in the hyperprior) in logit space corresponds to probability values being uniformly distributed between 0 and 1; we therefore wish to tell our model not to use larger values of σ_p, σ_o, or $\sigma_{\varepsilon ik}$, as these would squash all p_{ijk} to one of two modes, one at 0 and one at 1(25).

Markov Chain Monte Carlo Methods

medRxiv preprint doi: https://doi.org/10.1101/2020.04.11.20062018.this version posted April 24, 2020. The copyright holder for this preprint (which was not certified by peer review) is the author/funder, who has granted medRxiv a license to display the preprint in perpetuity.
This article is a US Government work. It is not subject to copyright under 17 USC 105 and is also made available for use under a CC0 license.

For all Bayesian models, we drew posterior samples using Stan (Stan Core Team 2018), which implements a No-U-Turn Sampler (a form of Markov Chain Monte Carlo), via its R interface RStan. We ran four replicate chains from random initial conditions for 2000 iterations, with the first 1000 iterations as a warmup/adaptation period. We saved the final 1000 iterations from each chain, giving us a total of 4000 posterior samples. We assessed convergence by inspecting trace plots and examining R□ and effective sample size (n_{eff}) statistics.

<u>Limit of detection (LOD)</u>

End-point titration has an approximate limit of detection set by the volume of the undilute sample deposited in each well. If all wells – including those containing undiluted sample – are negative and a Poisson single-hit model is used, the best guess is that the true titer lies somewhere below 1 $TCID_{50}$ / (volume of deposited sample). How far below is determined by the number of wells. For four wells, as was standard in our experiments, the first quarter $\log_{10}$ titer at which 0 wells is the most likely outcome is $10^{-0.5}$ $TCID_{50}$ per volume of sample. This is also the imputed Speaman-Karber titer in that case. Since we used samples of volume 0.1 mL, this corresponds to a value of $10^{0.5}$ $TCID_{50}$/mL. So although we do not use the Spearman-Karber method here (since we infer mean titers directly from the well data) we use that LOD value to plot samples for which no replicate had a positive well (since the posterior distribution in that case covers a wide-range of sub-threshold values).

Supplemental table

Table S1. Effect of decontamination method on SARS-CoV-2 viability. Results are reported as the median and upper- and lower-limits of the 95% credible interval of the estimated half-life, and time needed to reduce viable SARS-CoV-2 load by a factor of one thousand or one million, based on the posterior distribution of the exponential decay rate of the virus on different materials following different decontamination treatments.

medRxiv preprint doi: https://doi.org/10.1101/2020.04.11.20062018.this version posted April 24, 2020. The copyright holder for this preprint (which was not certified by peer review) is the author/funder, who has granted medRxiv a license to display the preprint in perpetuity.
This article is a US Government work. It is not subject to copyright under 17 USC 105 and is also made available for use under a CC0 license.

		**Half-life (min)**			**Time to one thousandth (min)**			**Time to one millionth (min)**		
**Treatment**	**Material**	**Median**	**2.5%**	**97.5%**	**Median**	**2.5%**	**97.5%**	**Median**	**2.5%**	**97.5%**
**Control**	N95 mask	78.7	66.1	90.4	784	659	901	1.57×10^3	1.32×10^3	1.8 × 10[illegible]
	Steel	290	244	327	2.89×10^3	2.43×10^3	3.26×10^3	5.77×10^3	4.86×10^3	6.53 [illegible] 10[illegible]
**Ethanol**	N95 mask	0.647	0.557	0.733	6.45	5.55	7.31	12.9	11.1	14.[illegible]
	Steel	1.08	0.895	1.26	10.8	8.92	12.5	21.6	17.8	2[illegible]
**Heat**	N95 mask	4.7	3.93	5.48	46.9	39.2	54.6	93.7	78.4	10[illegible]
	Steel	8.85	7.42	10.2	88.1	74	101	176	148	20[illegible]
**UV**	N95 mask	6.12	5.27	6.87	61	52.6	68.5	122	105	13[illegible]
	Steel	0.736	0.651	0.805	7.33	6.48	8.02	14.7	13	1[illegible]
**VHP**	N95 mask	0.999	0.83	1.14	9.95	8.27	11.3	19.9	16.5	22.[illegible]

medRxiv preprint doi: https://doi.org/10.1101/2020.04.11.20062018.this version posted April 24, 2020. The copyright holder for this preprint (which was not certified by peer review) is the author/funder, who has granted medRxiv a license to display the preprint in perpetuity.
This article is a US Government work. It is not subject to copyright under 17 USC 105 and is also made available for use under a CC0 license.

Steel	0.77	0.673	0.846	7.67	6.71	8.43	15.3	13.4	16.[illegible]

Code and data availability

Code and data to reproduce the Bayesian estimation results and produce corresponding figures are archived online at OSF: https://doi.org/10.17605/OSF.IO/mkg9b and available on Github: https://github.com/dylanhmorris/n95-decontamination

medRxiv preprint doi: https://doi.org/10.1101/2020.04.11.20062018.this version posted April 24, 2020. The copyright holder for this preprint (which was not certified by peer review) is the author/funder, who has granted medRxiv a license to display the preprint in perpetuity. This article is a US Government work. It is not subject to copyright under 17 USC 105 and is also made available for use under a CC0 license.

Acknowledgements

We would like to thank Madison Hebner, Julia Port, Kimberly Meade-White, Irene Offei Owusu, Victoria Avanzato and Lizzette Perez-Perez for excellent technical assistance. This research was supported by the Intramural Research Program of the National Institute of Allergy and Infectious Diseases (NIAID), National Institutes of Health (NIH). JOL-S and AG were supported by the Defense Advanced Research Projects Agency DARPA PREEMPT # D18AC00031 and the UCLA AIDS Institute and Charity Treks, and JOL-S was supported by the U.S. National Science Foundation (DEB-1557022), the Strategic Environmental Research and Development Program (SERDP, RC□2635) of the U.S. Department of Defense. Names of specific vendors, manufacturers, or products are included for public health and informational purposes; inclusion does not imply endorsement of the vendors, manufacturers, or products by the US Department of Health and Human Services.

Supplemental references

1. Ranney ML, Griffeth V, Jha AK. Critical Supply Shortages - The Need for Ventilators and Personal Protective Equipment during the Covid-19 Pandemic. N Engl J Med. 2020 Mar 25.

2. McMichael TM, Currie DW, Clark S, Pogosjans S, Kay M, Schwartz NG, et al. Epidemiology of Covid-19 in a Long-Term Care Facility in King County, Washington. N Engl J Med. 2020 Mar 27.

3. Batelle. Final Report for the Bioquell Hydrogen Peroxide Vapor (HPV) Decontamination for Reuse of N95 Respirators. 2016 [cited; Available from: https://www.fda.gov/media/136386/download

4. Fisher EM, Shaffer RE. A method to determine the available UV-C dose for the decontamination of filtering facepiece respirators. J Appl Microbiol. 2011 Jan;110(1):287-95.

5. Heimbuch BK, Wallace WH, Kinney K, Lumley AE, Wu CY, Woo MH, et al. A pandemic influenza preparedness study: use of energetic methods to decontaminate filtering facepiece respirators contaminated with H1N1 aerosols and droplets. Am J Infect Control. 2011 Feb;39(1):e1-9.

6. Lin TH, Tang FC, Hung PC, Hua ZC, Lai CY. Relative survival of Bacillus subtilis spores loaded on filtering facepiece respirators after five decontamination methods. Indoor Air. 2018 May 31.

medRxiv preprint doi: https://doi.org/10.1101/2020.04.11.20062018.this version posted April 24, 2020. The copyright holder for this preprint (which was not certified by peer review) is the author/funder, who has granted medRxiv a license to display the preprint in perpetuity. This article is a US Government work. It is not subject to copyright under 17 USC 105 and is also made available for use under a CC0 license.

7. Avilash Cramer ET, Sherryl H Yu, Mitchell Galanek, Edward Lamere, Ju Li, Rajiv Gupta, Michael P Short. disposable N95 masks pass qualitative fit-test but have decreases filtration efficiency after cobalt-60 gamma irradiation. MedRxiv.

8. Lin TH, Chen CC, Huang SH, Kuo CW, Lai CY, Lin WY. Filter quality of electret masks in filtering 14.6-594 nm aerosol particles: Effects of five decontamination methods. PLoS One. 2017;12(10):e0186217.

9. (NIOSH) TNIfOSaH. Decontamination and Reuse of Filtering Facepiece Respirators . 2020 [cited 2020 4/5/2020]; Available from: https://www.cdc.gov/coronavirus/2019-ncov/hcp/ppe-strategy/decontamination-reuse-respirators.html

10. CDC. Recommended Guidance for Extended Use and Limited Reuse of N95 Filtering Facepiece Respirators in Healthcare Settings. 2020 [cited; Available from: https://www.cdc.gov/niosh/topics/hcwcontrols/recommendedguidanceextuse.html

11. Ong SWX, Tan YK, Chia PY, Lee TH, Ng OT, Wong MSY, et al. Air, Surface Environmental, and Personal Protective Equipment Contamination by Severe Acute Respiratory Syndrome Coronavirus 2 (SARS-CoV-2) From a Symptomatic Patient. JAMA. 2020 Mar 4.

12. van Doremalen N, Bushmaker T, Morris DH, Holbrook MG, Gamble A, Williamson BN, et al. Aerosol and Surface Stability of SARS-CoV-2 as Compared with SARS-CoV-1. N Engl J Med. 2020 Mar 17.

13. Heimbuch BK, Kinney K, Lumley AE, Harnish DA, Bergman M, Wander JD. Cleaning of filtering facepiece respirators contaminated with mucin and Staphylococcus aureus. Am J Infect Control. 2014 Mar;42(3):265-70.

14. Mills D, Harnish DA, Lawrence C, Sandoval-Powers M, Heimbuch BK. Ultraviolet germicidal irradiation of influenza-contaminated N95 filtering facepiece respirators. Am J Infect Control. 2018 Jul;46(7):e49-e55.

15. Viscusi DJ, Bergman MS, Eimer BC, Shaffer RE. Evaluation of five decontamination methods for filtering facepiece respirators. Ann Occup Hyg. 2009 Nov;53(8):815-27.

medRxiv preprint doi: https://doi.org/10.1101/2020.04.11.20062018.this version posted April 24, 2020. The copyright holder for this preprint
(which was not certified by peer review) is the author/funder, who has granted medRxiv a license to display the preprint in perpetuity.
This article is a US Government work. It is not subject to copyright under 17 USC 105 and is also made available
for use under a CC0 license.

16. CDC. Chemical Disinfectants, Guideline for Disinfection and Sterilization in Healthcare Facilities. 2008 [cited; Available from: https://www.cdc.gov/infectioncontrol/guidelines/disinfection/disinfection-methods/chemical.html#Hydrogen

17. FDA. N95 Respirators and Surgical Masks (Face Masks). 2020 [cited 2020 4/5/2020]; Available from: https://www.fda.gov/medical-devices/personal-protective-equipment-infection-control/n95-respirators-and-surgical-masks-face-masks

18. Administration OSaH. Temporary Enforcement Guidance - Healthcare Respiratory Protection Annual Fit-Testing for N95 Filtering Facepieces During the COVID-19 Outbreak. 2020 [cited; Available from: https://www.osha.gov/memos/2020-03-14/temporary-enforcement-guidance-healthcare-respiratory-protection-annual-fit

19. Administration OSaH. User Seal Check Procedures (Mandatory). 2020 [cited 2020 April 11]; Available from: https://www.osha.gov/laws-regs/regulations/standardnumber/1910/1910.134AppB1

20. Administration OSaH. Respirator Fit Testing [WWW Document]. U. S. Dep. Labo. 2012 [cited; Available from: https://www.osha.gov/video/respiratory_protection/fittesting_transcript.html (accessed 4.10.20).

21. Holshue ML, DeBolt C, Lindquist S, Lofy KH, Wiesman J, Bruce H, et al. First Case of 2019 Novel Coronavirus in the United States. N Engl J Med. 2020 Jan 31.

22. van Doremalen N, Bushmaker T, Munster VJ. Stability of Middle East respiratory syndrome coronavirus (MERS-CoV) under different environmental conditions. Euro Surveill. 2013 Sep 19;18(38).

23. Brownie C, Statt J, Bauman P, Buczynski G, Skjolaas K, Lee D, et al. Estimating viral titres in solutions with low viral loads. Biologicals. 2011 Jul;39(4):224-30.

24. Gelman A. Bayesian data analysis. Third edition. ed. Boca Raton: CRC Press; 2014.

25. Northrup JM, Gerber BD. A comment on priors for Bayesian occupancy models. PLoS One. 2018;13(2):e0192819.
